# Laser-Stimulated Fluorescence in Paleontology

**DOI:** 10.1371/journal.pone.0125923

**Published:** 2015-05-27

**Authors:** Thomas G. Kaye, Amanda R. Falk, Michael Pittman, Paul C. Sereno, Larry D. Martin, David A. Burnham, Enpu Gong, Xing Xu, Yinan Wang

**Affiliations:** 1 Burke Museum of Natural History and Culture, Seattle, Washington, United States of America; 2 Southwestern Oklahoma State University, Department of Biology, Weatherford, Oklahoma, United States of America; 3 Vertebrate Palaeontology Laboratory, Department of Earth Sciences, University of Hong Kong, Pokfulam, Hong Kong, China; 4 Department of Organismal Biology and Anatomy, University of Chicago, Chicago, Illinois, United States of America; 5 Division of Vertebrate Paleontology, Biodiversity Institute, Natural History Museum, University of Kansas, Lawrence, Kansas, United States of America; 6 Division of Vertebrate Paleontology, Biodiversity Institute, Natural History Museum, University of Kansas, Lawrence, Kansas, United States of America; 7 Department of Geology, Northeastern University, Shenyang, Liaoning, China; 8 Institute of Vertebrate Paleontology and Paleoanthropology, Beijing, China; 9 1111 Army Navy Drive, Arlington, Virginia, United States of America; University of Zurich, SWITZERLAND

## Abstract

Fluorescence using ultraviolet (UV) light has seen increased use as a tool in paleontology over the last decade. Laser-stimulated fluorescence (LSF) is a next generation technique that is emerging as a way to fluoresce paleontological specimens that remain dark under typical UV. A laser’s ability to concentrate very high flux rates both at the macroscopic and microscopic levels results in specimens fluorescing in ways a standard UV bulb cannot induce. Presented here are five paleontological case histories that illustrate the technique across a broad range of specimens and scales. Novel uses such as back-lighting opaque specimens to reveal detail and detection of specimens completely obscured by matrix are highlighted in these examples. The recent cost reductions in medium-power short wavelength lasers and use of standard photographic filters has now made this technique widely accessible to researchers. This technology has the potential to automate multiple aspects of paleontology, including preparation and sorting of microfossils. This represents a highly cost-effective way to address paleontology's preparatory bottleneck.

## Introduction

Highlighting and identifying fossilized structures can be difficult whether it is bone, soft tissue such as skin, muscle and internal organs, or integument such as scales and feathers. Historically, multiple methods have been used to highlight structures for photography, including cross-lighting, polarized light [1], camera filters, and ultraviolet (UV) light [2–6]. Cross-lighting can highlight structures that are difficult to see in direct light. Polarized light can help to enhance image contrast. UV light is capable of causing minerals (e.g. bone [hydroxyapatite]) to fluoresce, and can even highlight soft tissue to some extent [7]. This paper describes a next-generation method of fluorescing minerals using specific wavelengths of light produced by a laser and corresponding imaging through the use of laser-blocking longpass camera filters (see Methods). This method is herein named Laser-stimulated fluorescence (LSF).

For many decades UV light has been used at night to find and collect fluorescent mineral specimens, which are prized for their wide variation in color [8]. The field of biology has made tremendous scientific advances through the use of laser-induced fluorescence mostly through the widespread use of confocal laser microscopes [9–12]. In paleontology, UV light has seen increasing use in recent years where the resulting fluorescence can often reveal structures and patterns not seen under white light [13, 14]. The typical UV light source consists of commonly available standard fluorescent lamps with low wattage and a wavelength of 364 nanometers (nm) [7]. Greater amounts of UV flux on the specimen will cause fluorescent minerals to become more conspicuous, allowing for easier photographic documentation, sometimes with the aid of UV filters (e.g. Hoya brand) [6]. The limited variety of detectable fluorescence in fossils has been a primary limitation in the past using standard UV bulbs [14].

The technique presented here utilizes laser illumination to stimulate fluorescence which offers an order of magnitude improvement in the signal-to-noise ratio over standard UV light. The irradiance of a 20 watt UV fluorescent lamp is about 510 milliwatts per square centimeter (mWcm-2) at a distance of 20 centimeters from the target [15], but the irradiance of a ½ watt laser is on the order of 4000–8000 mWcm-2 [16]. This results in detectable fluorescence of many hard-to-fluoresce mineral types which typically remain dark under standard UV. This advantage can be leveraged when other factors are accounted for. For instance, matching the correct laser line with one of the specimen’s absorption bands provides more effective excitation of the fluorescence in a sample. Furthermore, using the right optical filter that matches one of the fluorescence bands of the specimen would improve contrast in the fluorescence image.

Each color of laser emits a different wavelength of light, which will excite fossils and matrix from different rock units in different ways, as the case histories that follow will indicate. Again, LSF techniques depend on the wavelength of light used, the filter used, and the inherent fluorescent properties of the rocks under study. The exact methodology used, therefore, is going to vary depending on these properties.

Laser-induced fluorescence imaging performed through confocal laser-scanning microscopy (CLSM) has been used in micropaleontology to study the morphology and cellular anatomy of fossils *in situ*, at micron-scale resolution, and even in three-dimensions [17–19]. LSF is a simplified and more accessible version of CLSM that uses simpler laser beam scanning and data acquisition systems, and lacks a confocal hole. However, the LSF technique provides its own unique advantages in studying macroscopic paleontological specimens including the compactness and low cost of its setup, its fast data acquisition rate and its high sensitivity compared to UV-stimulated fluorescence. The purpose of this paper is to describe the laser-stimulated fluorescence (LSF) imaging technique and to formalize its use in paleontology in the hope that new and more efficient modes of discovery will be possible.

## Methods

Laser-stimulated fluorescence (LSF) imaging is a versatile observational technique that has a multitude of paleontological applications. Both automated and manual systems can be used to scan or otherwise observe fossils under laser illumination. A series of common steps apply to any LSF work, these are detailed below.

Laser light is concentrated on a specimen either as a point source for microscopic work, as a divergent light cone for smaller-sized specimens (with the aid of a laser diffuser), or a collimated beam (in which all light rays are parallel) is raster scanned over very large specimens. Since the laser is very bright, it must be blocked with an appropriate filter that still allows the longer wave fluorescence signal to pass through. Proper precautions using laser-blocking protective glasses and manufacturer’s safety protocol should be followed.

The equipment used with this methodology depends on the exact wavelength of light produced by the laser. Specialized light-blocking longpass filters, often used in astronomy, are best-suited for these methods. These particular filters will allow all wavelengths of light longer than a certain wavelength to pass through the filter, however, it will stop all shorter wavelengths. For instance, a red-orange longpass filter (LP580, MidOpt) will allow 91–95% of light between the wavelengths of 600–1100 nm, however, the transmission sharply decreases between 600–520 nm, and by 510 nm, no light passes through the filter (www.midopt.com). A 477 nm blue laser would be efficiently blocked by this filter, but will still allow imaging of longer fluorescent wavelengths. The laser wavelengths and filters for each particular specimen were chosen experimentally via the trial and error method, a procedure that we believe is reasonable given the simplicity of this technique. The setup parameters for each of the five case histories presented in this study are given in Table 1.

**Table 1:** Setup parameters for five case histories.

Case history	Laser wavelength / nm	Lasercolor	Laser wattage /mW	Filter	Wavelength blocked / nm
1	532	green	300	O-54	<540
2	532	green	300	O-54	<540
	457	blue	500	Y-48	<480
3	532	green	150	O-54	<540
4	532	green	250	O-54	<540
5	408	violet	500	Y-44	<440
	532	green	500	O-54	<540

Listing of laser wavelength and wattage plus filter type for five case histories.

Standard UV bulbs can be used in addition to lasers in order to cover a broad range of the light spectrum. Imaging is done in both UVA from 315–400 nm and UVB from 280–315 nm. When working with UV light, photographs can be taken both with and without filters due to the low UV sensitivity of digital camera CCD (charge-coupled device) chips.

No special digital cameras are needed to photograph specimens using laser fluorescence. Typically, digital single lens reflex cameras (DSLRs) capable of manual time exposures (e.g. Nikon D610) with either wide angle or macro lenses are sufficient. Ideally, the photography should be done in a darkroom, basement, or office without windows or with blackout curtains, as any influence of natural light will reduce the clarity of the fluorescence. The use of a tripod is necessary, as the exposure time during photography is typically long—up to several minutes, although this may not be the case for close macro photography. The aperture setting (f-value) should be as low as possible for long-exposure shots.

Multiple types of laser light sources can be used. The more powerful the laser, the better and brighter the fluorescence. For the experiments outlined here, class III lasers in the 300–500 mW category were used. These were well below the threshold that results in radiation damage to the specimens studied. A lab laser, which plugs into the wall and is fairly static, and a high-powered laser pointer that runs off of CR123A lithium batteries, have both been used successfully depending on the locality of the specimen. The benefit of using a lab laser is that it can be used for hours at a time without overheating. It is typically used for precision work and photographing larger specimens. A high-power laser pointer is more portable and adjustable than a lab laser, however it can only be used for ~5 minutes, or else it will overheat and become damaged. If the photographer knows what f-value and shutter speeds are necessary for photography, a laser pointer can be used to great effect. It is excellent for macro photography in the field due to its portability.

**Fig 1:**
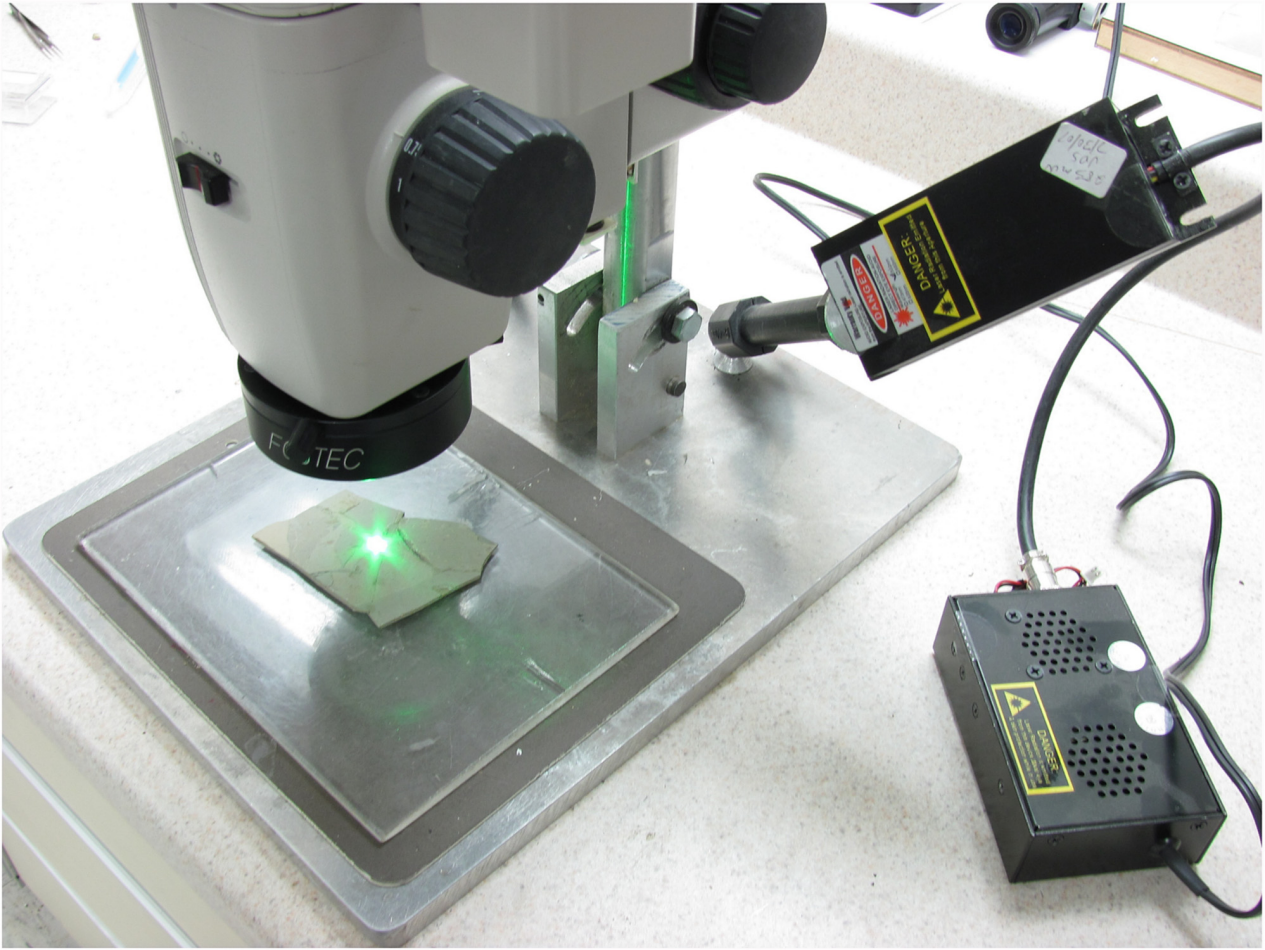
Laser mounted on a stereo microscope. A 532 nm green laser mounted to the side of a stereo microscope. The blocking filter is mounted in front of the objective lens. No diffuser is used here for maximum effect with a smaller spot.

A laboratory setup for table-top-sized specimens would typically hold the laser on a fixed mount (Fig 1). The laser itself emits a collimated beam, which results in only a small dot of illumination. This beam can be used as is for maximum flux or be expanded using a diffuser (ARF used a 20-degree diffraction diffuser from Thorlabs). The smaller the angle of the diffuser, the better—i.e. 20 degrees would be better than 50 degrees, as it restricts the beam to a narrower angle and results in a brighter and smaller area of illumination, even if the laser is placed further away from the specimen. The laser should illuminate as much of the specimen as possible, and the diffuser’s cone angle changes the area covered by the laser depending on the laser-to-specimen distance.

**Fig 2:**
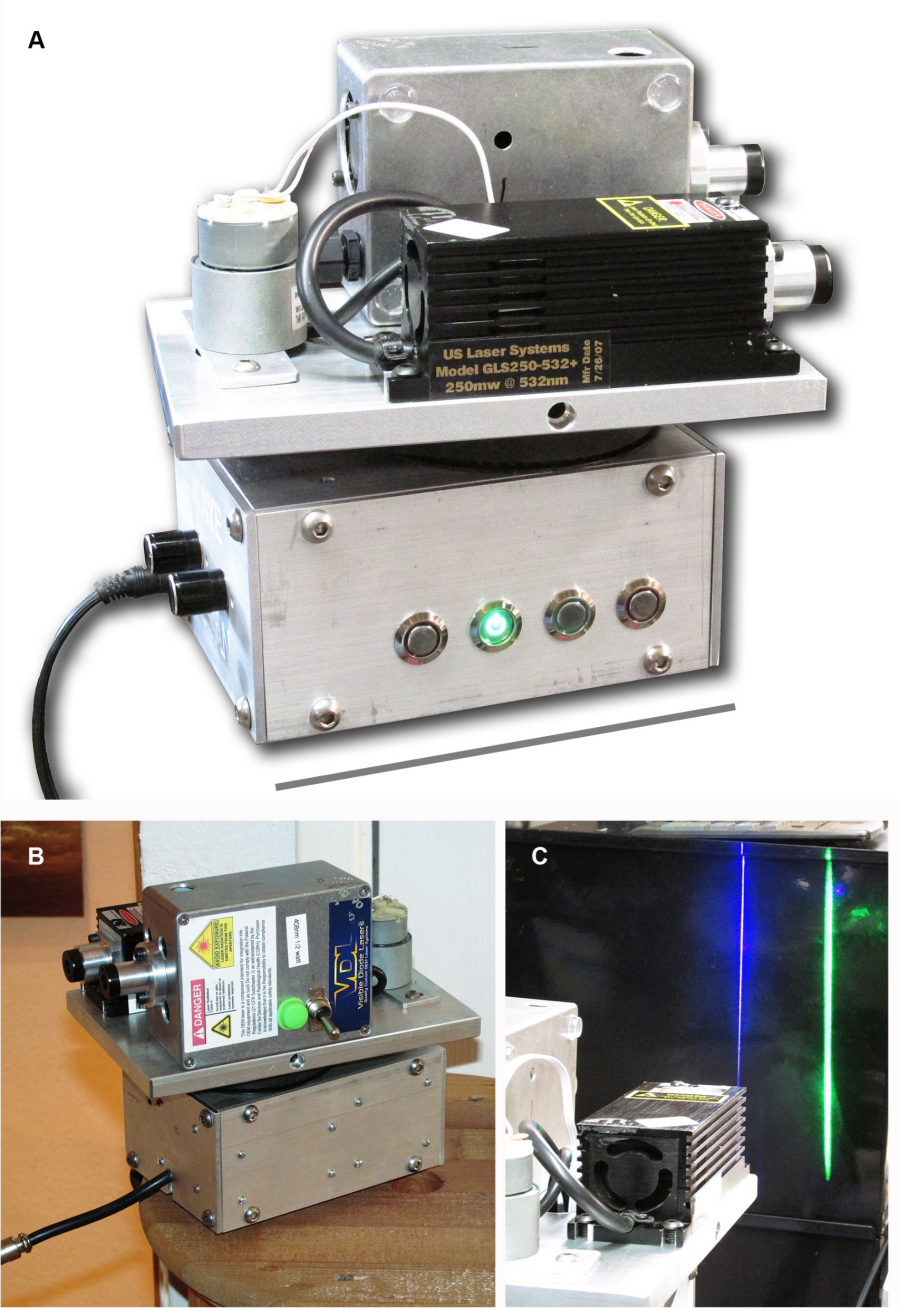
Laser scanning system. A, Custom-built laser scanning device. B, Blue and green laser modules are mounted onto the scanning plate. A variable speed DC motor scans the plate back and forth laterally and is adjustable for degrees covered. C, Removable line lenses convert the laser spot to a more diffuse vertical line. Scale bar in Fig 2A equals 15 cm.

Larger specimens can be scanned using a custom device (Fig 2A and 2B). A Powel laser line lens projects a laser line in the Y direction that evenly distributes the laser energy over the length of the line (Fig 2C). A motor scans the entire assembly in the X direction (Fig 2A and 2B). This allows specimens of almost any size to be imaged. The exposure time of the DSLR camera should cover one or more of the X direction scans of the specimen.

For a microscope setup, the collimated laser beam is directed through one of the illumination ports or projected directly onto the specimen. The emitted light, laser and fluorescence, comes back through the microscope’s optical train where a longpass filter is placed either before the objective lens or internally in a filter slot to block the intense laser light. The fluorescence can then be observed and photographed in detail.

Specimen sources for each case history:

Case history 1: Burke Museum of Natural History and Culture, UWBM 103073—feather from Green River Fm.; UWBM 103074—feather from Parachute Member of Green River Fm.

Case history 2: Department of Land and Resources of Liaoning Province, LVH 0026—fish specimen from Jiufotang Fm. [20]

Case history 3: UWBM 103075—microfossils from Brule Fm.; UWBM 103076—microfossils from Hell Creek Fm.

Case history 4: Gobero specimen housed in the University of Chicago Research Collection, G1B2—juvenile female skeleton from mid-Holocene lake deposits

Case history 5: Institute of Vertebrate Paleontology and Paleoanthropology, Beijing, China, IVPP V13320—*Microraptor* skull from Jiufotang Fm. [21]

## Case Histories

The setup parameters for all case histories are given in Table 1 of the Methods section.

### Case history 1: Silhouette illumination of carbon films

Feathers and other carbonized structures—like ichthyosaur body outlines and fossil leaves—offer unusual insight into certain soft tissues preserved in the fossil record. Usual methods of reflected light microscopy and scanning electron microscopes (SEM) detail surface topography only. The vastly preferred method for microscopy uses transmitted rather than reflected light, but carbon films are typically never thin-sectioned to allow for back illumination. Completely carbonized organic matter is typically not fluorescent, but immature carbonaceous matter of fossilized organisms may fluoresce [18]. In this case, and in similar cases encountered so far, the background matrix is composed of a highly fluorescent mineral that silhouettes the carbon films so that they show up in stark contrast to the matrix around them—the fluorescence intensity of the matrix contrasts with the apparent non-fluorescence of the carbonaceous matter. However, the latter can fluoresce to a small degree in some specimens.

**Fig 3:**
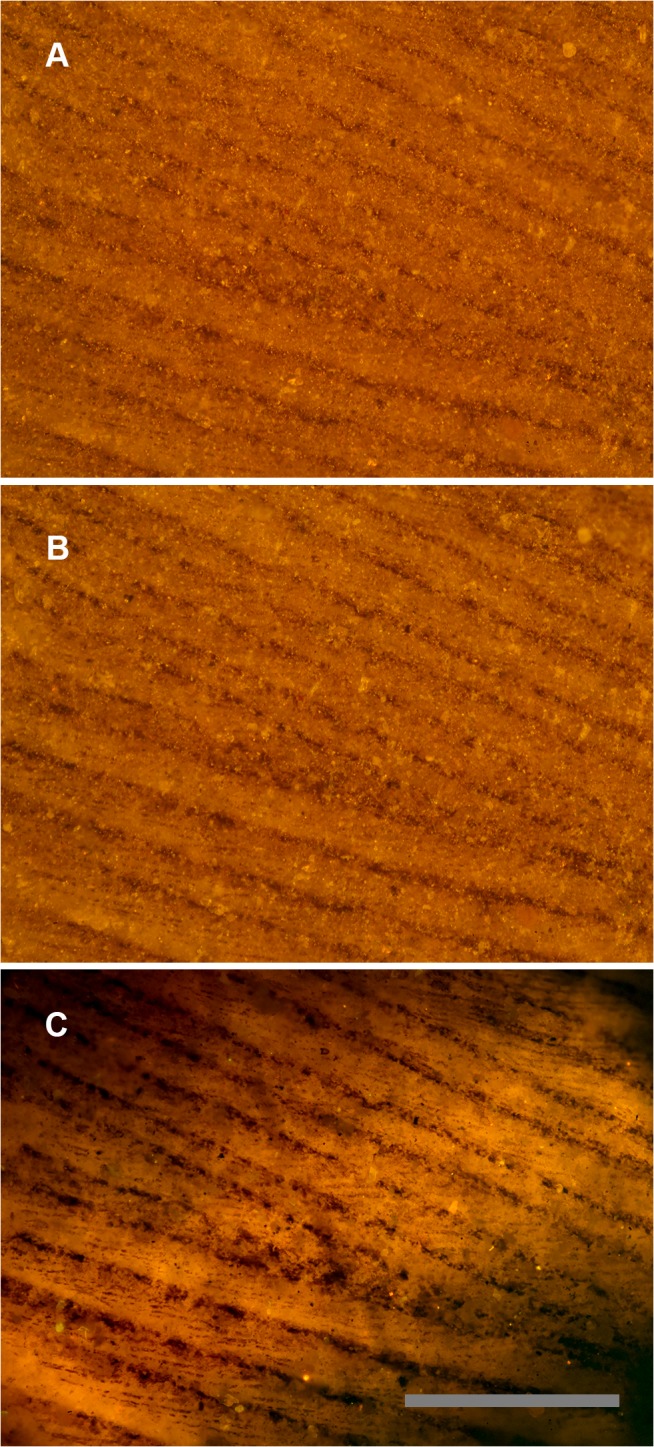
Feather under reflected and matrix fluoresced illumination. Green River Formation feather using identical images under different lighting conditions. A, Reflected light microscopy, only barbs are visible. B, Polarized light, some traces of barbules. C, Laser-stimulated fluorescence of matrix behind the carbon film backlights the feather and renders barbules visible across the entire field of view. Scale bar 0.5 mm.

**Fig 4:**
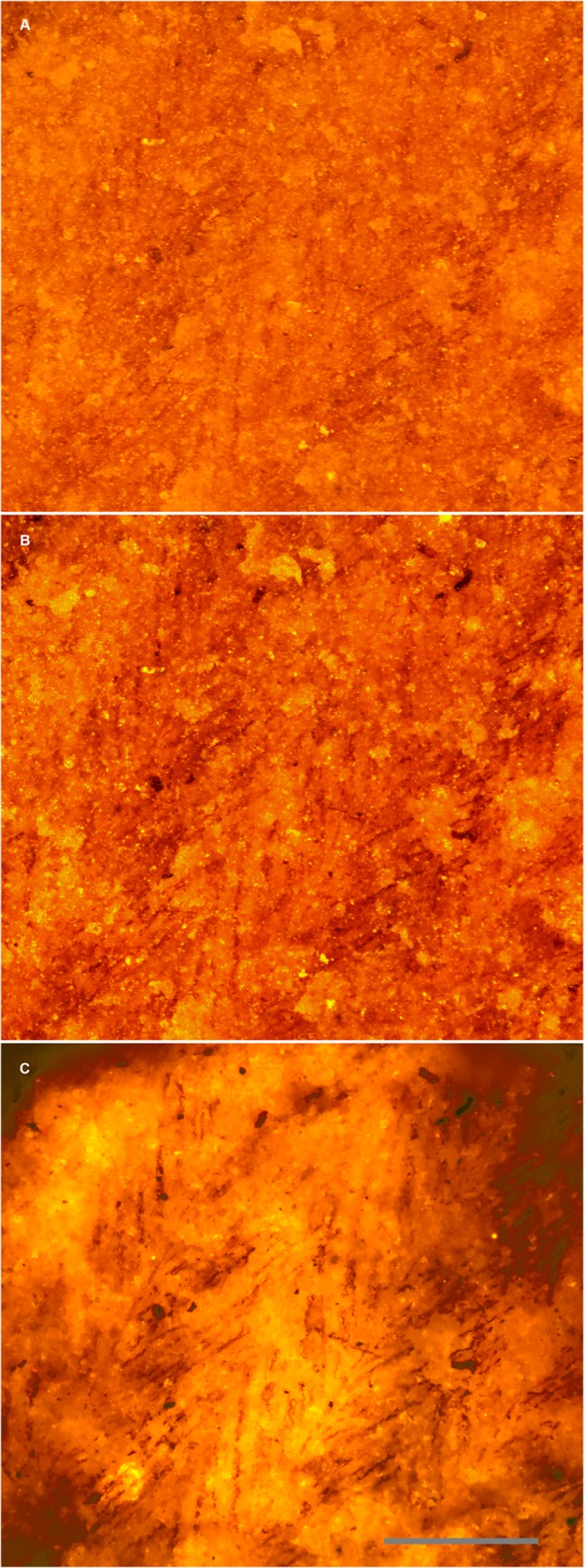
Feather structure comparison using white light, polarized and laser illumination. A second Green River Formation feather specimen under: A, white light, B, polarized light, and C, laser illumination. Scale bar 0.2 mm.

Fig 3 shows a feather from the Green River Formation under the microscope in reflected (Fig 3A) and polarized light (Fig 3B). It clearly shows the barbs, but no barbules are immediately apparent. Fig 3C shows the same field using a smaller laser spot to fluoresce the matrix under the carbon film. It is clear that barbules are in fact present and widely distributed in this specimen. Multiple specimens from the same formations showed these results (Fig 4). Back side illumination also removes topography from the image and makes partially buried barbules visible.

### Case history 2: Microscopic imaging of specimens fluorescing beneath the specimen surface

**Fig 5:**
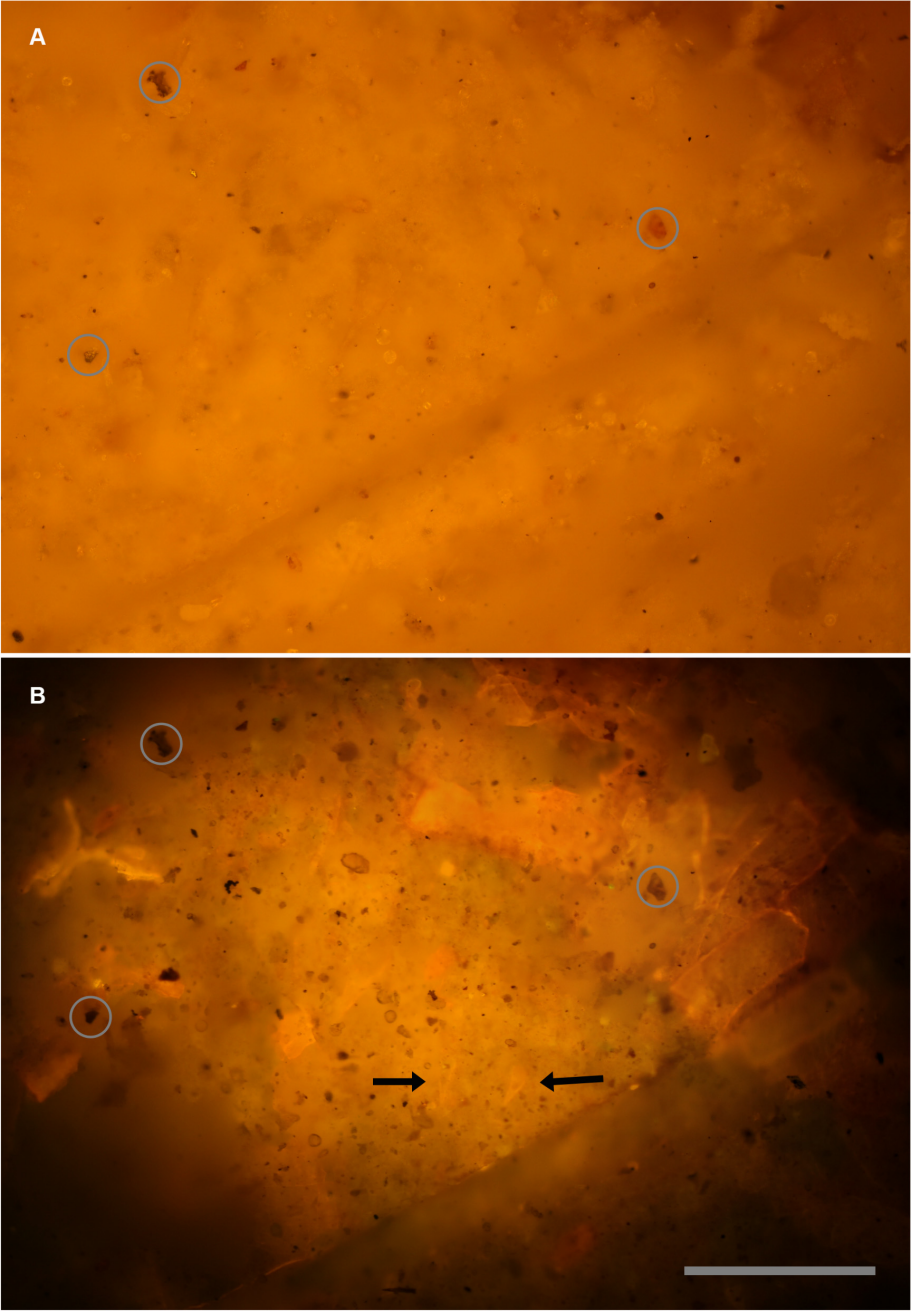
Micro-imaging—visible light vs. **laser fluorescence.** Identical views of the same field. A, Reflected white light image of a purposely non-descript field. B, Same field illuminated with a 532 nm laser. Some features are at the specimen’s surface, but lack color contrast, others are visible within the matrix of the specimen. Black arrows indicate teeth found coincidentally in this image. Circles indicate reference landmarks. Scale bar 0.15 mm.

The previous case history showed how laser light can penetrate into the matrix and backlight surface specimens. This example shows how the same technique can illuminate and visualize specimens completely covered in the matrix or transparent at the surface. Fig 5 compares the white light and fluorescence results revealing new details on the surface of the specimen and within its matrix.

**Fig 6:**
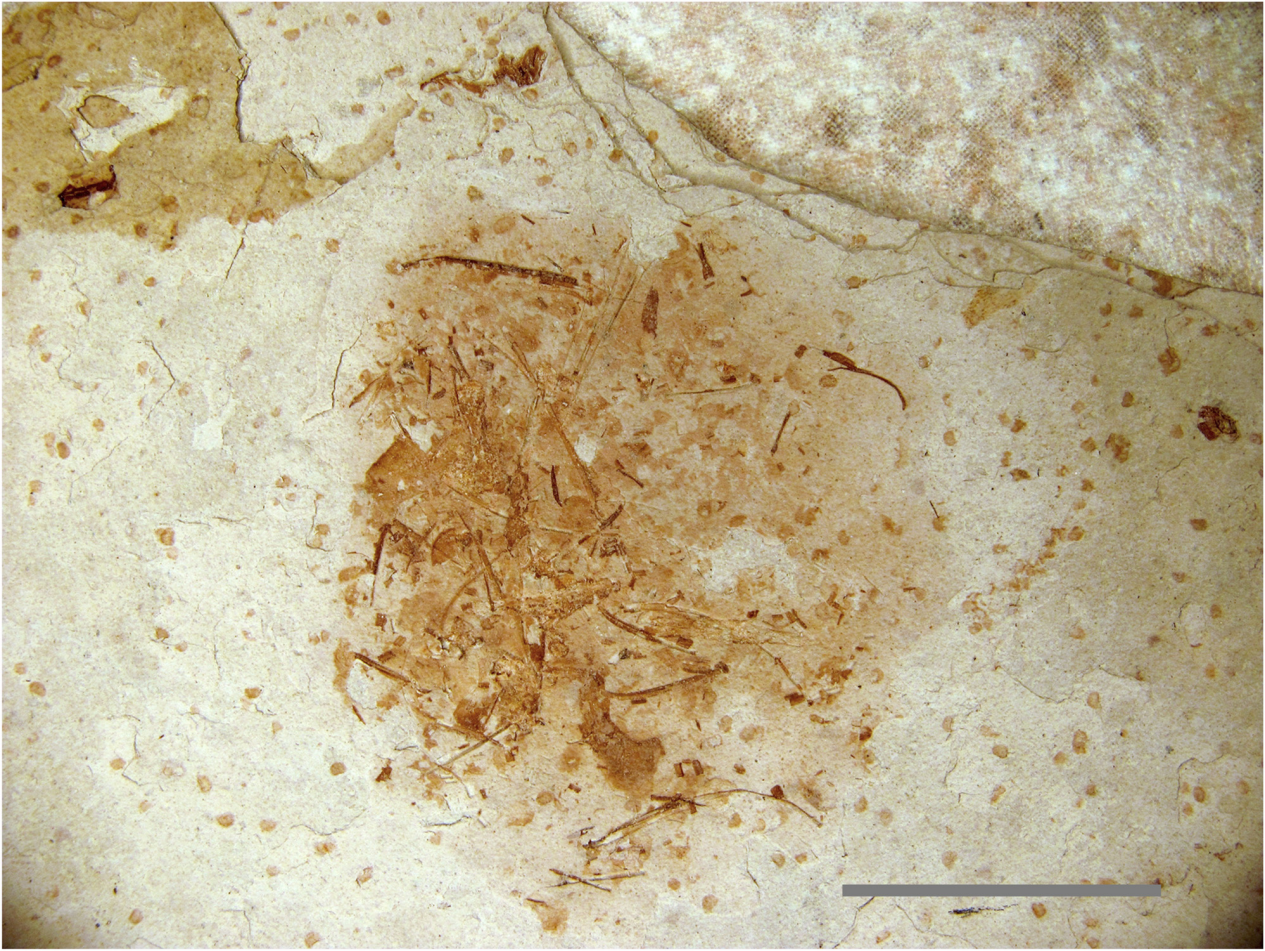
Unidentified Liaoning fossil specimen. An unidentifiable specimen from a Liaoning rock slab containing a *Microraptor* specimen (LVH 0026). No diagnostic bones are visible on the specimen surface, but laser penetration into the matrix induced fluorescence in multiple teeth and scales, making the identification of a fish possible. Scale bar 1 cm.

**Fig 7:**
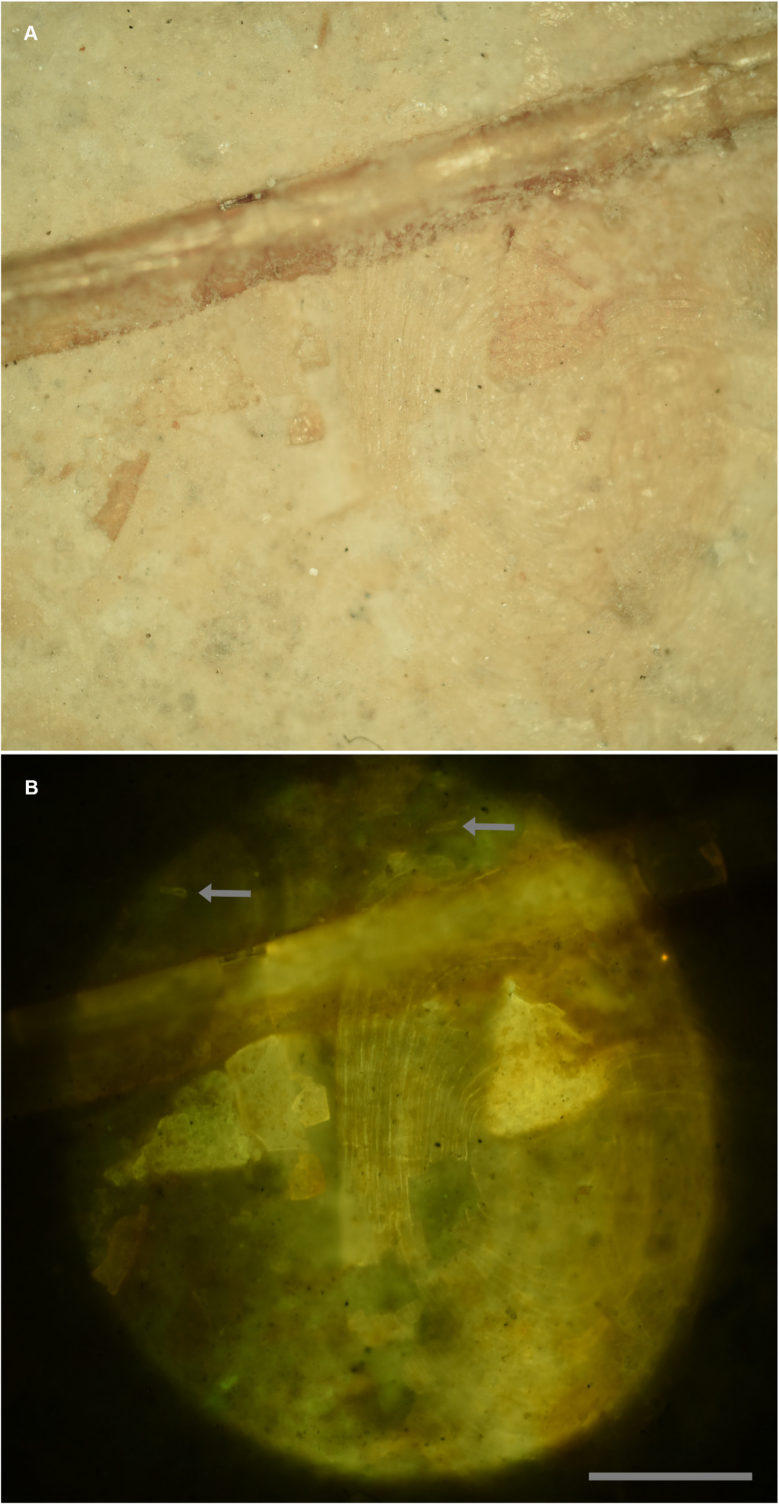
Details enhanced with laser fluorescence. A, White light photo. B, Fluoresced with a 457 nm blue laser. Fish scale on the surface is translucent and barely perceivable under white light. Growth rings on the scale are revealed under fluorescence and can be counted. Bone fragments are brought out in sharp detail. Arrows point to teeth in the matrix. Scale bar 0.5 mm.

A very small specimen (Fig 6) was discovered on the same slab as a *Microraptor* specimen (Theropoda: Dromaeosauridae [20]) discovered in Liaoning province, China (LVH 0026). The visible bones were not sufficient to identify the specimen, so it was submitted for laser analysis as a last resort. Laser fluorescence identified the specimen as a fish within minutes. In this case the hydroxyapatite in the bones and teeth fluoresced at a higher intensity than the surrounding matrix. The higher intensity fluorescent reaction of the specimen, in comparison to the matrix, revealed teeth below the surface and transparent scales on the surface that were virtually invisible in reflected light (Fig 7). Note that the bone fragment on the right in Fig 7 actually lies under the scale. Laser fluorescence of the formerly translucent scale shows enough detail to count the scale’s growth rings.

**Fig 8:**
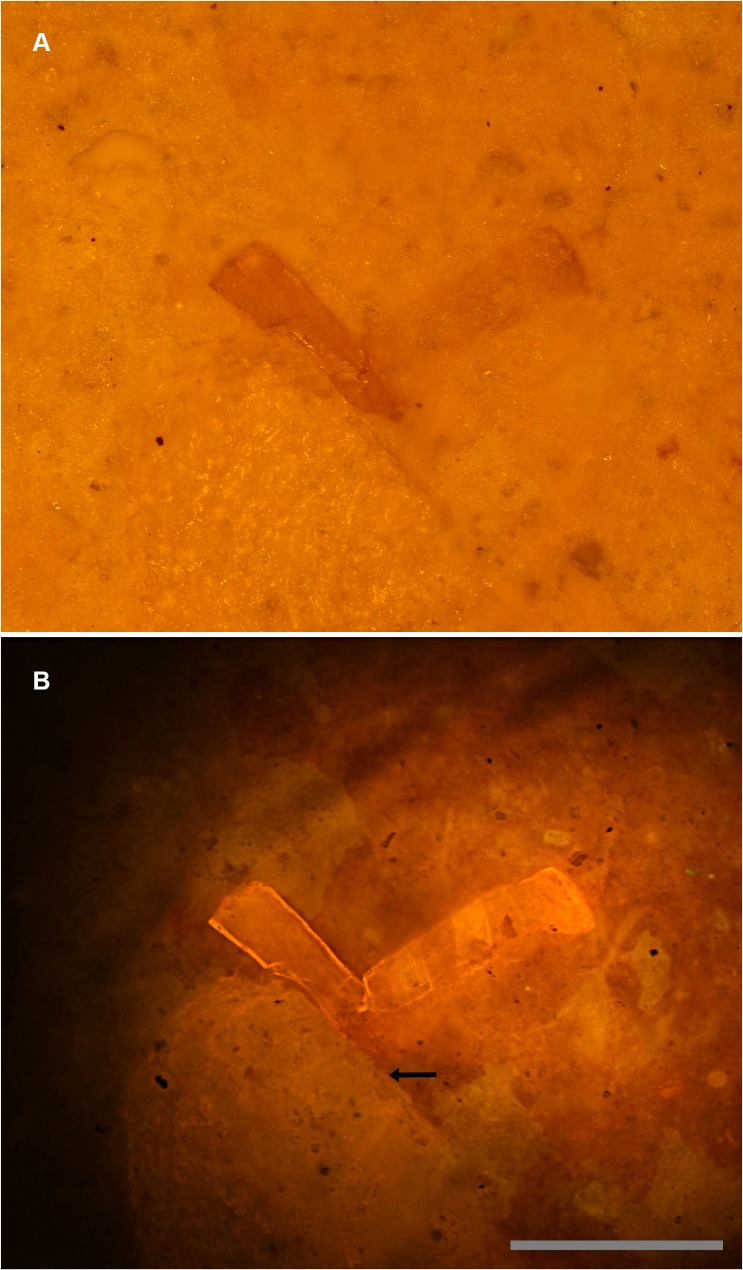
Sub-surface imaging. A, White light micrograph shows the bone fragment on the right entombed within matrix. B, Specimen under laser fluorescence. Photograph shows a high level of detail invisible under white light. Note that another much larger fragment also becomes visible (arrow). Scale bar 0.5 mm.

The laser has a unique ability not seen using lower intensity UV fluorescent bulbs, which is to penetrate a short distance into the matrix and fluoresce buried specimens (Fig 8A and 8B). The top image shows a partially buried bone fragment on the right in white light. Fig 8B shows the margins of the fragment in stark detail. The ability of the intense laser to penetrate into the matrix to reveal hidden specimens is a technique previously only available through regular X-rays and expensive computer tomography (CT) scans [22]. However, this laser property has already been utilized via laser-Raman and CLSM methods to characterize microscopic specimens buried within matrix [18, 19, 23]. The laser has limited penetration, but for specimens buried in finely-laminated sedimentary rocks, material may be limited to the first lamination, as was the case with this specimen. This makes the technique valuable for this and potentially many other Liaoning specimens. Specimens with a uniform matrix that is translucent to laser, fluorescent and scattered light are especially ideal for this technique. This is because the laser beam will be experience less distortion from inhomogeneities in the matrix and will achieve a greater penetration depth.

### Case history 3: Automated sorting of micro-fossils

In testing, fossil teeth and bone from formations such as the Eocene Chadron Formation and Oligocene Brule Formation (White River Group) fluoresce well under fluorescent bulb UV light, but small specimens from the Late Cretaceous Lance and Hell Creek Formations do not. Lance and Hell Creek microfossils are prized because of the rarity of mammals and small dinosaurs from these formations. Typical methodologies for recovering these specimens utilize screen washing or anthill collecting. Ants select specific grain sizes to build an anthill over their colony exit and inadvertently concentrate small fossils (usually no more than a few mm across). In either case, the resulting concentrate is historically manually scanned under a microscope by an operator, which in virtually all cases takes weeks to years to go through just a few kilograms of concentrate.

**Fig 9:**
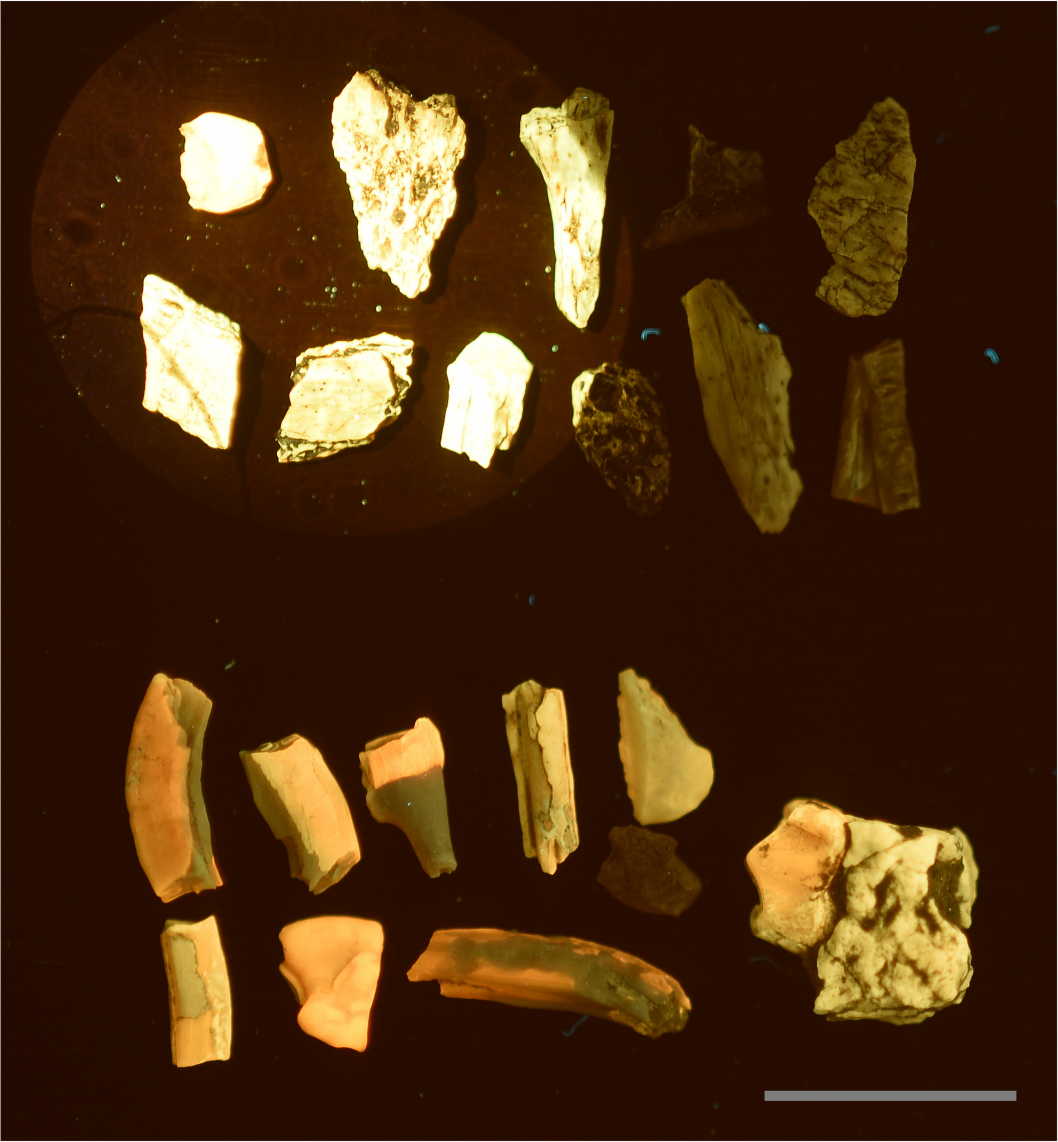
Fluorescent black light vs. **blue laser.** A direct comparison between a 15 watt fluorescent UV light illuminating all the fossils at a distance of 7cm, and a 447nm 500mw laser stimulating the specimens in the upper left corner. A, Specimens from the Lance Formation exhibit very low reactivity under fluorescent UVA bulbs. B, Specimens from the White River Formation typically fluoresce very well. This demonstrates that the intensity of laser stimulation can influence low reactivity specimens to fluorescence several orders of magnitude better than specimens known to fluoresce well under UV bulbs. Scale bar 1 cm.

Experiments were conducted on Lance and Hell Creek specimens using a non-dispersed 300 mW, 532nm green laser beam to induce maximum flux. Results indicated that specimens from both formations would indeed fluoresce under these conditions (Fig 9). A stereo microscope was set up with a longpass filter attached to the objective, and a laser beam was directed to the middle of the visual field (Fig 1). A one layer deep pan of concentrate was then raster scanned by hand under the microscope in search of specimens that fluoresced. Given the high contrast of the fluorescing specimens, these could be identified very quickly compared to the conventional approach of identifying specimens based on their color, texture and morphology. This inexpensive setup allows concentrate to be worked through much faster than conventional microscopic sorting.

**Fig 10:**
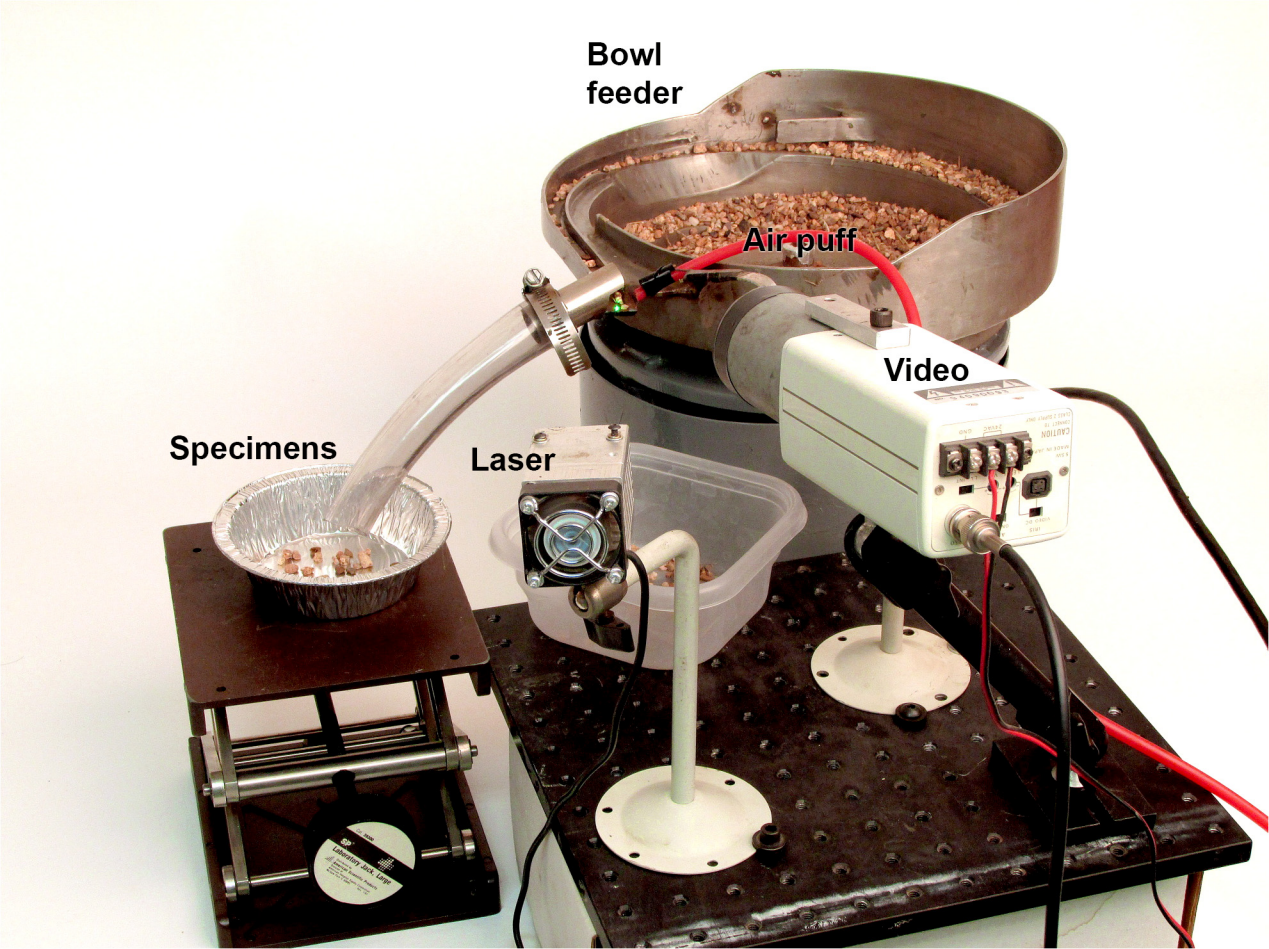
Automated fossil sorter. Proof-of-concept prototype automated micro-fossil picker. The feeder bowl guides a stream of matrix under the laser while a video camera detects ‘blobs’ of a certain size and brightness. Fluorescing fossils are guided down a tube into a tray by a puff of compressed air.

The success of the manual method was followed by an experimental setup to automatically sort fluorescing fossils from concentrate without human intervention [24]. The entire experimental apparatus consisted of an industrial feeder bowl, green laser, video camera, computer-controlled air puff, and two bins for reject material and candidate specimens (Fig 10). See Supplemental Information for a more detailed description of the system (S1 Fig).

A vibratory bowl feeder was set up to feed a continuous narrow stream of material that was completely illuminated by the laser. A video camera with an appropriate longpass filter viewed the material moving under the laser. When the brightness of a specified group of pixels exceeded a preset threshold, a computer, linked to the video camera, would trigger a short puff of air that would move the specimen into the side tube so it can fall into the “super concentrate” bin.

Experiments showed that this machine could effectively process about 1–2 kilograms per hour. The resulting concentrate comprised of ~20–50% fossil material with the rest being made up of fluorescing organics and extraneous grains that were blown in with the fossils during the air puff. This is believed to be the first ever automated sorting system in paleontology. Widespread implementation and use of this technology could greatly expand our knowledge of ancient microfauna.

### Case history 4: In situ analysis with minimal equipment

**Fig 11:**
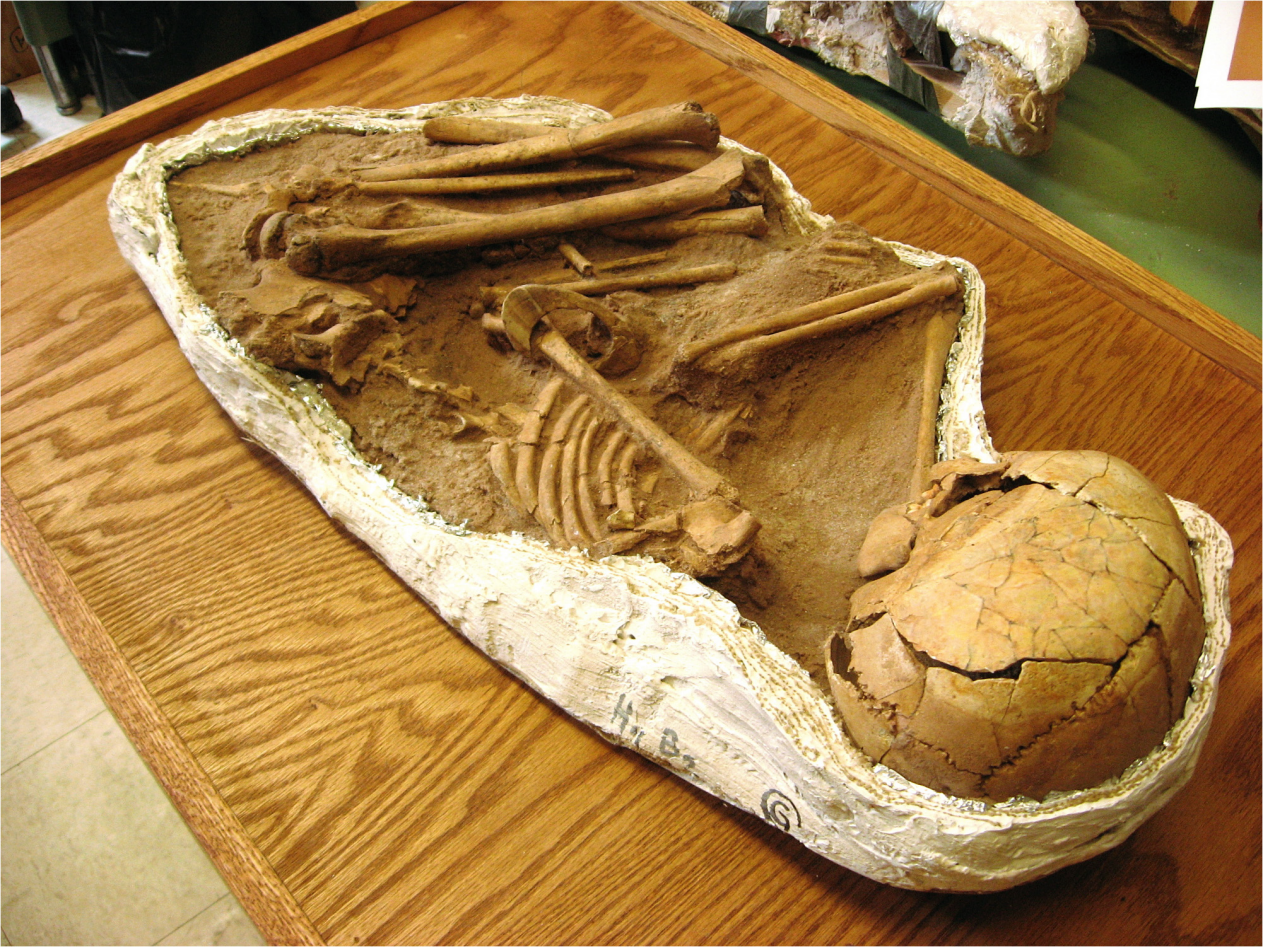
*In situ* investigation. A mid-Holocene-aged Gobero skeleton of a small girl preserved wearing an arm bracelet (G1B2; ~2835 B.C.E.) [25]. Due to the impossibility of removing the bracelet, analysis required portable, non-invasive techniques.

This example focuses on the use of minimal equipment for *in situ* evaluation of a difficult-to-examine specimen. A mid-Holocene-aged skeleton of a young girl from the Sahara desert of Gobero, Niger (Fig 11) was found with an intact bracelet around her arm (G1B2; ~2835 B.C.E.) [25]. It was not possible to move the skeleton or remove the artifact.

**Fig 12:**
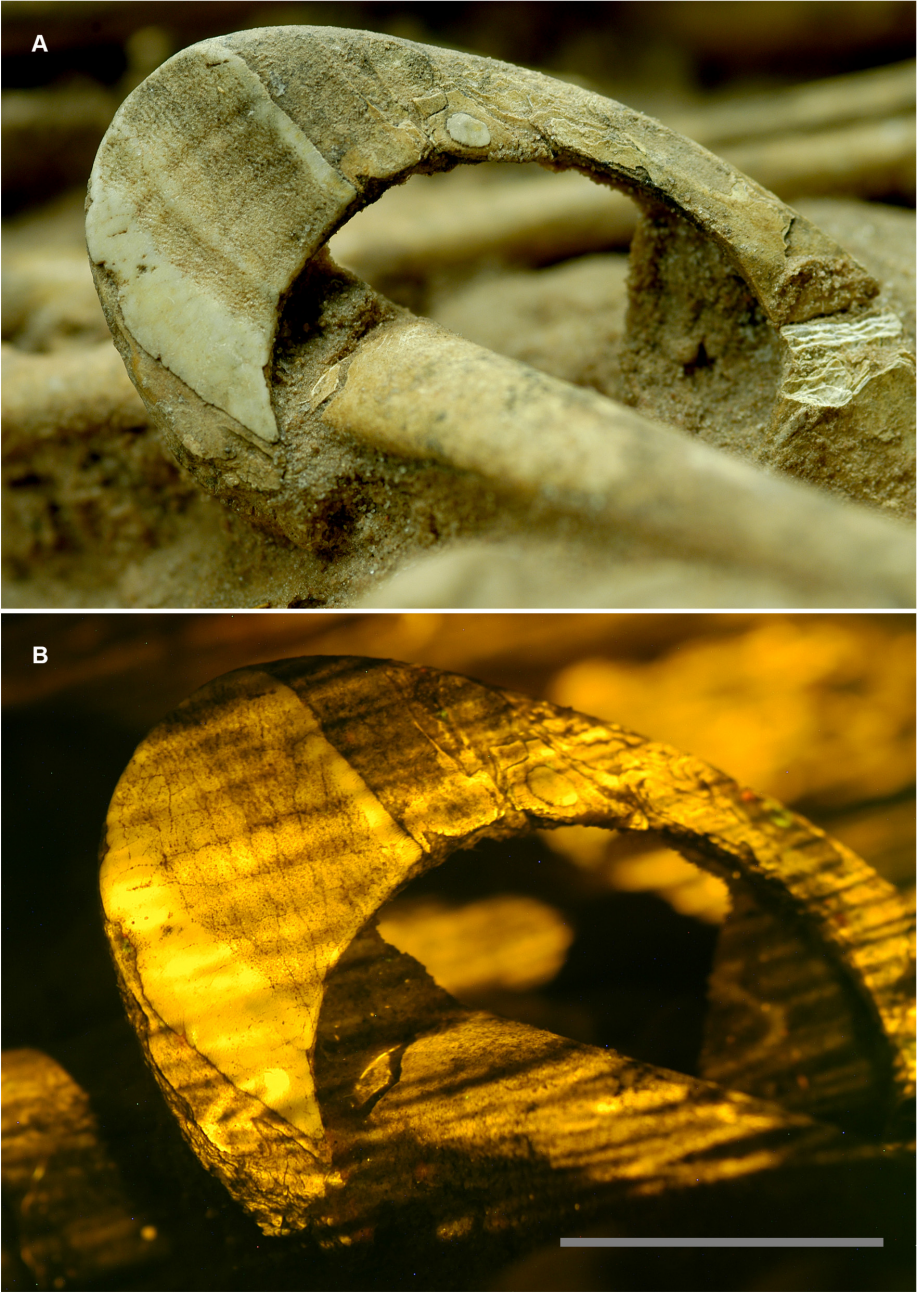
Bracelet fluorescing under laser stimulation. A, The bracelet (G1B2 [25]) under normal light, and B, The bracelet fluorescing under a hand-scanned laser. The cracking pattern in the upper left corner is only visible under fluorescence and aided in the identification of the bracelet material as hippopotamus tooth. Scale bar 2 cm.

The circumstances surrounding the specimen necessitated a rapid investigation, and proper laser scanning equipment was not available. A 532 nm green laser was raster scanned by hand over the artifact in a dark room during a timed exposure of a DSLR camera fitted with a yellow longpass filter. The resulting fluorescence exposed a crack pattern (Fig 12) that was one of several lines of evidence used to identify the bracelet material as coming from a hippopotamus tusk (hippo ivory).

### Case history 5: Using LSF to spot potential composite “Two faced” *Microraptor* specimen

**Fig 13:**
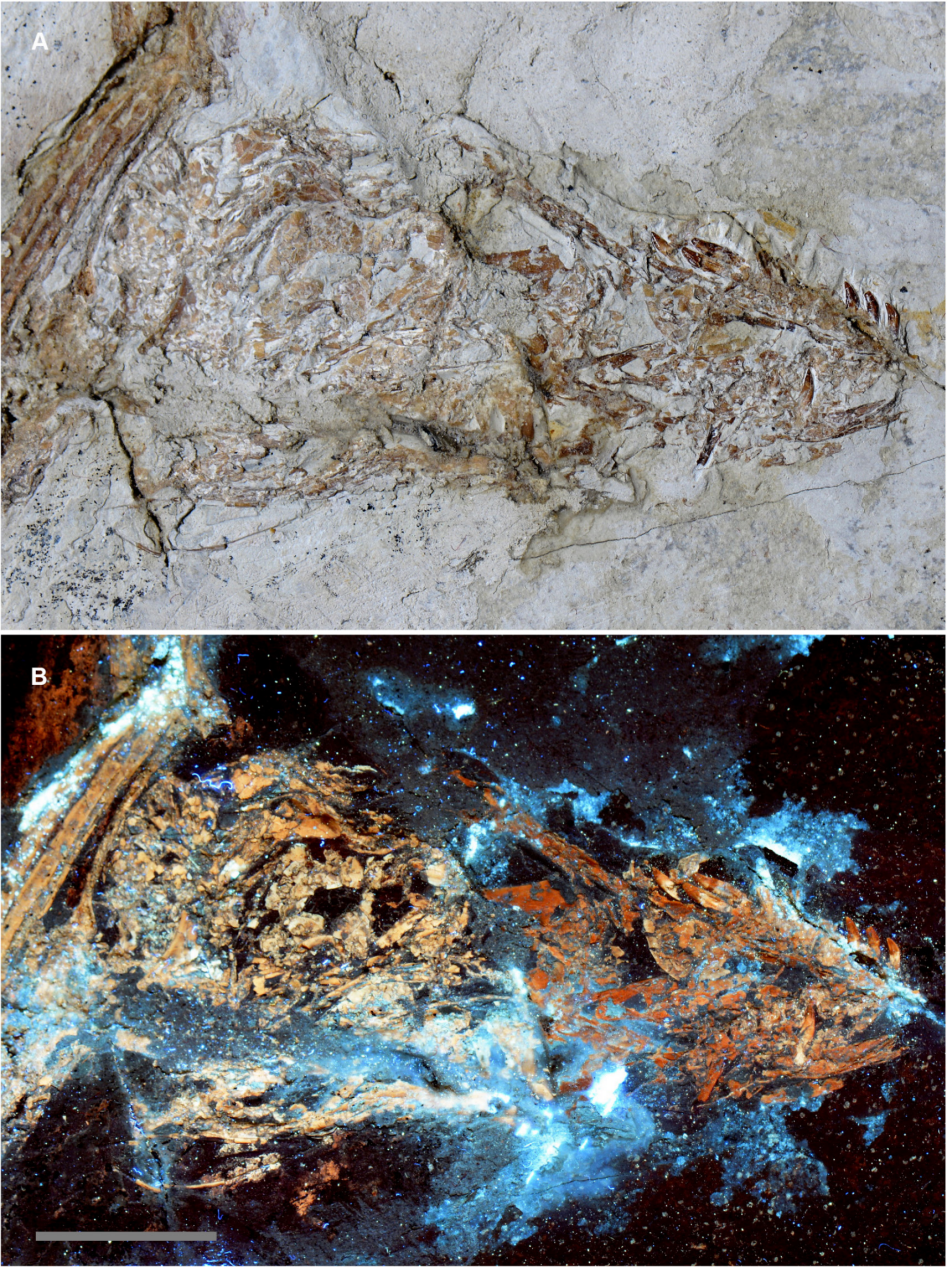
Is the skull of *Microraptor* IVPP V13320 a composite? A, the skull of IVPP V13320 under white light conditions shows subtle color differences in the bone across a break in the slab—darker bone proximally and lighter bone distally. B, Under laser light stimulation, the bone fluoresces with the same color pattern observed under white light conditions (see A) indicating that the color differences relate to differences in fossil mineralogy. The latter indicates that the skull is a composite specimen, but it is also possible—but less likely—that the pattern observed reflects variable depositional and taphonomic conditions. Scale bar 1 cm. S2 Fig is a labelled version of this figure (see Supporting Information).

The skull of a *Microraptor* specimen (IVPP V13320 [21]) was first examined under white light conditions. Although there are subtle color differences in the fossil, nothing unusual stands out on first inspection (Fig 13A). Subsequent imaging using LSF reveals dramatic differences in fluorescence between the proximal and distal portions of the skull prompting further investigation (Fig 13B).

These results could relate to different skull ossification centers as theropods show postnatal elongation of the rostrum [26–28]. However, this hypothesis is discounted because the fluorescence pattern does not follow bone boundaries, but a break in the slab (Fig 13B). These mineralogical differences may instead reflect variability in the specimen’s depositional environment (source minerals and pore water composition) and taphonomic history (microbial activity and diagenesis). At a finer scale within the proximal and distal portions of the skull, cartilage and different bone types all fluoresce with different colors e.g. the scleral ring and some premaxillary tooth roots have a lighter fluorescence color than the rest of the darker-colored proximal portion of the skull (Fig 13B).

Both the proximal and distal skull portions belong to *Microraptor* (see Supporting Information for more details, S2 Fig), but a shared dorsal margin for the frontal and most of the teeth as well as a shared ventral margin for the suspected postorbital, splenial and prearticular, suggests that the ventral margin of the proximal skull portion is strangely positioned dorsally. Taken together with the fluorescence results, these observations indicate that the skull of IVPP V13320 might be a composite specimen; this will need to be confirmed by future work using more immobile, higher-cost chemical mapping techniques such as synchrotron rapid scanning X-ray fluorescence (SRS-XRF) [29]. LSF can improve the efficiency with which potential composite fossils are identified because it is more mobile than CT scanning and produces more vivid fluorescence images than UV light stimulation (see Introduction and Methods) [30–33]. However, as mentioned, LSF is also a useful tool for identifying differences in bone type which can reveal information about an animal’s bone ossification centers and ontogeny. LSF can also uncover heterogeneity in a specimen’s depositional environment and taphonomic history.

## Discussion

There is a short list of non-destructive techniques that are both affordable and accessible for use in paleontology. Laser-stimulated fluorescence (LSF) is a new addition to this list. It provides an instantaneous, non-invasive, geochemical fingerprint of fossilized bone, soft tissue, integument and the surrounding matrix. LSF is also valuable for uncovering anomalies in specimens that direct the user towards more detailed investigations using other methods such as Raman spectroscopy—LSF is not presently quantifiable as to the precise elemental or molecular nature of the material. In addition to the aforementioned fossilized structures, anomalies can also relate to the use of glue, differences in matrix and bone composition, and the potential presence of composite skeletons. With higher sensitivity than UV lamp-based fluorescence imaging, and the compactness and low cost of the setup, LSF promises to become a diagnostic paleontological technique for everyday use. LSF imaging therefore has great potential for use in future descriptive work, particularly of fossils from well-preserved assemblages (konservat-lagerstätten) like those of the Mesozoic of northeastern China, which can have incomplete provenance information.

In the examples given in this paper, new information was revealed using relatively inexpensive and rapid techniques that would otherwise have not been discovered without the use of much more expensive and/or time-intensive methods. Setups start from around US$500 and process within seconds to minutes making LSF easily accessible.

The ability to look into the matrix for hidden specimens with simple techniques like the ones described here (Case history 2), was previously only possible using X-rays, CT scans, CLSM and RAMAN which have high cost and low accessibility [18, 23]. Micro-CT has certainly revolutionized the way small specimens are dealt with, but in virtually all cases the specimens are already known and CT is not likely to be used as a basic discovery process due to its expense. Silicates can often diffuse a concentrated laser beam several millimeters into the matrix. Future experiments may quantify the depth of matrix penetration using more powerful lasers and may extend the detection depth further than expected. This technique is particularly useful for fossils that are too large to fit onto a standard microscope stage or into regular CT scanners. It is also useful for fossils with an insufficient density contrast with their host matrix, which cannot produce meaningful CT images. The technique can therefore help to bridge a number of important knowledge gaps relating to such fossils.

LSF can also be used in a novel way as a light source behind non-fluorescing features, such as carbon films (Figs 3 and 4). These soft-tissue remains have become progressively more important and are improving our understanding of fossil morphologies, but the techniques to investigate them have been slow to develop. Small details in carbon films can be missed using reflective light microscopy, however, the use of LSF will often backlight carbonized films both on and slightly below the surface. This clarifies the image and creates extra contrast that makes carbonized structures easier to see, especially smaller structures (e.g. the feather barbules in Figs 3 and 4). If LSF is adopted as a standard investigative tool, we may be able to further investigate carbonized films and other non-fluorescing soft tissues. The backlighting technique can also highlight material defects, which could be phylogenetically informative e.g. the distinctive cracking pattern of hippo ivory (Case history 4). Such defects may also reveal useful biomechanical and behavioral information if they relate to tooth, tusk and bone loading and wear.

Multi-spectral imaging with different wavelengths highlights the fact that various minerals and compounds fluoresce with different intensity, according to wavelength. This is an important property as it may not always be desirable to fluoresce the entire specimen. In the case of the auto-picker, only bone specimens were of interest and the green laser was an ideal choice. Had a method such as UVB been employed the bone fragments would have been overwhelmed by other common minerals and organics, which also fluoresce under UVB, defeating the purpose of the machine.

## Conclusions

The advent of low cost, high power lasers including blue, green and violet wavelengths, means that the laser-stimulated fluorescence (LSF) technique can become widely available. The filters can be sourced from the photography industry or astronomy suppliers again at reasonable cost. The compactness and portability of the system combined with its efficiency in scanning large and extremely small fossils fills a gap in our ability to quickly and easily analyze specimens. It provides instant geochemical comparisons over a wide range of specimens, including specimens that previously exhibited very low fluorescence activity. At the microscopic level, LSF has already proven its worth in revealing unseen details in many specimens. The use of fluorescence is already in common use in multiple subdisciplines of biology, and is clearly a technique that is useful in paleontology as well. We have only scratched the surface of the ability of fluorescence to contribute to paleontology. LSF already holds a ‘first’ in the automation of micro-fossil sorting and this could theoretically be extended into automated preparation of specimens which is the single biggest bottleneck in paleontology. Overall, LSF is a *discovery* process geared toward uncovering unusual features of interest for follow up examination with more specialized tools.

## Supporting Information

S1 Fig Diagrammatic illustration of automatic sorting machine. (TIF)

S2 Fig The skull of Microraptor IVPP V13320, with anatomical labels. A, white light image. B, Fluorescent image. Scale bars 1 cm. Abbreviations: pm? t, suspected premaxillary teeth; m? t; probable maxillary teeth; m?, suspected maxilla; o, orbit; sr (?), (potential) scleral ring; n?, suspected nasal; f, frontal; po?, possible postorbital; sp?, probable splenial; pa?, possible prearticular.
(TIF)

S1 Text (DOCX)
