# Supplementary figures and images for: Laser-Stimulated Fluorescence in Paleontology
Source: PLoS One. 2015 May 27;10(5):e0125923. doi: 10.1371/journal.pone.0125923 (PMC4446324; doi:10.1371/journal.pone.0125923)

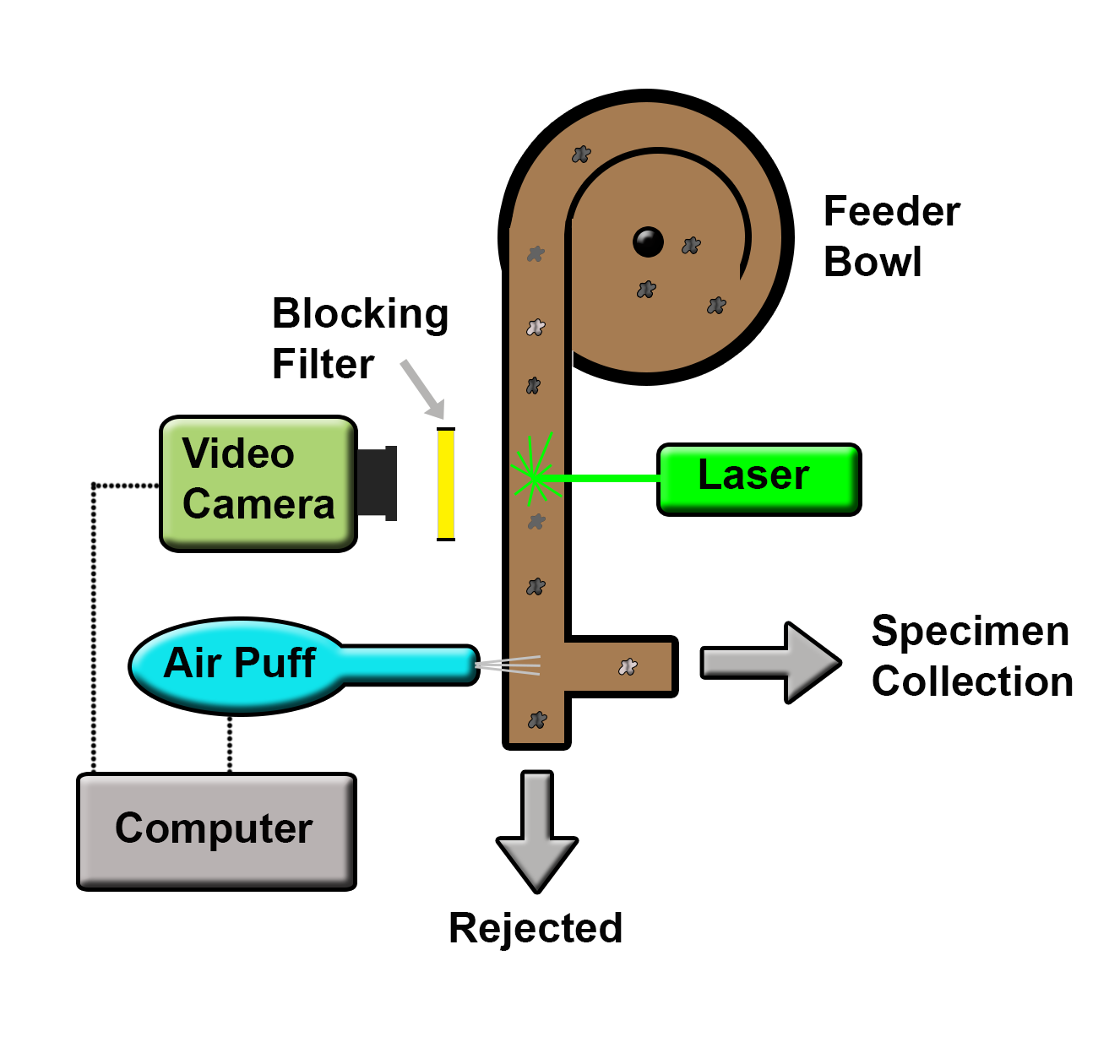

Supplement: S1 Fig — (TIF) [file pone.0125923.s001.tif]

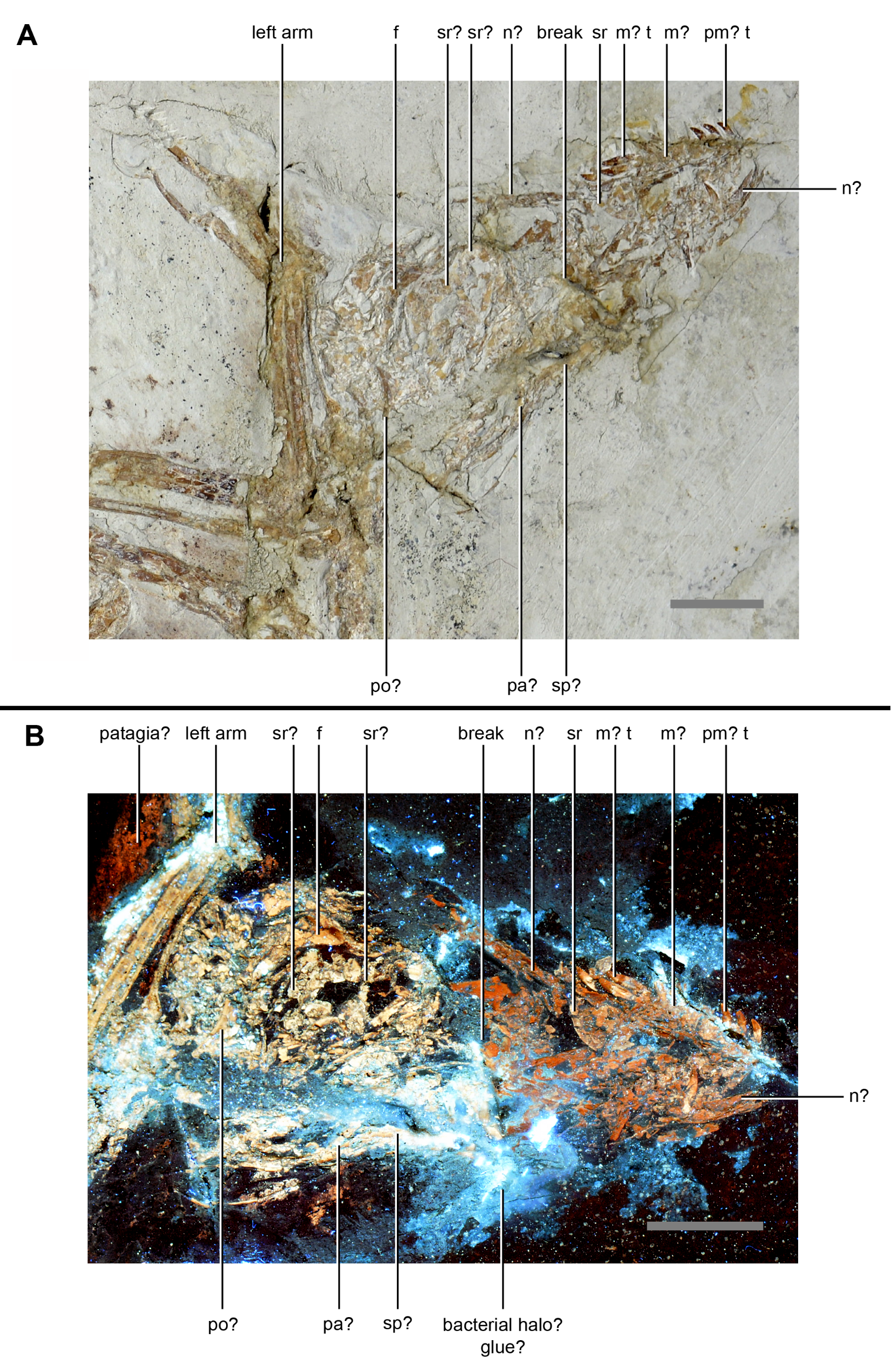

Supplement: S2 Fig — A, white light image. B, Fluorescent image. Scale bars 1 cm. Abbreviations: pm? t, suspected premaxillary teeth; m? t; probable maxillary teeth; m?, suspected maxilla; o, orbit; sr (?), (potential) scleral ring; n?, suspected nasal; f, frontal; po?, possible postorbital; sp?, probable splenial; pa?, possible prearticular. (TIF) [file pone.0125923.s002.tif]
