# Supplementary material for: Laser-Stimulated Fluorescence in Paleontology
Source: PLoS One. 2015 May 27;10(5):e0125923. doi: 10.1371/journal.pone.0125923 (PMC4446324; doi:10.1371/journal.pone.0125923)
Supplement: S1 Text — (DOCX) [file pone.0125923.s003.docx]

**Imaging Methodology Details**

**Case histories 1 and 2:**All images fluoresced using a 300 milliwatt (mw) 532 nanometer (nm) green laser. A Zeiss Axiomat was custom retrofitted with the laser for through lens reflected light imaging. A 540 nm longpass filter was used for all images similar to Hoya brand O54. Multiple images were focus stacked, contrast enhanced and sharpened using Adobe Photoshop.

**Case history 3:**An industrial bowl feeder was modified to feed a fine stream of anthill gravel down a narrow trough. The stream was illuminated with a collimated 150 milliwatt 532 nm green laser. A video camera output was converted to a webcam feed for processing by the software. The video was focused through a 540 nm longpass filter on the point of the laser illuminated stream. The video feed was then processed frame by frame using RoboRealm software. The software triggered a pulse of air to deflect the specimen into a side container whenever a pre-determined size and brightness threshold was passed.

**Case history 4:**All images were taken with a Nikon D100 camera and Nikkor 105 mm macro lens. A 150 milliwatt laser with a collimated beam was hand-scanned over the specimen. The camera was fitted with a 540 nm longpass filter. Final images were contrast enhanced and sharpened in Adobe Photoshop.

**Case history 5:**The specimen was horizontally scanned using a 500 milliwatt 447 nm laser incorporating a Laser Line Optics Canada line lens to produce a vertical laser line. The laser was mounted on a custom made scanning platform. The camera used was a Nikon D610 with a 50 mm Nikkor lens. A ‘Y2’ 470 nm longpass filter was used to block the laser.

**Image Processing**

Fluorescence data greatly benefits from post-processing the images using software such as Adobe Photoshop. Often slight differences between areas of the specimen are not readily obvious. The typical DSLR camera records images through red, green and blue filters which can each produce different data. Separating each of the colors into mono images allows for direct comparison to identify fluorescent signals. Working with each mono image individually and recombining them in different ways (mono-R to G etc.) allows for enhancement of difficult features. Recombination of separate RGB (red, green, blue) images can also color features of interest in brighter shades making them easier to communicate.

Typically, color equalization is the first step to bring out all the information in the picture. Equalization is followed by color saturation and then adjustments to brightness, contrast and gamma, as required. In all cases, in order to avoid artifacts and human influence, all pixels in an image are subjected to the same processes. Post-processing fluorescent images should always be considered for best results and easiest interpretation.

**The taxonomic status of the skull of IVPP V12330**

The skull portion proximal to the break is identified as *Microraptor* based on the absence of anterior serrations on all teeth and posterior teeth that are constricted at their base [[1](#_ENREF_1)] (S2 Fig.). The portion of the skull distal to the break has no discernible dromaeosaurid or deinonychosaurian synapomorphies, but the postcranial skeleton – which shares the same fluorescence color as the distal skull portion – is identified as *Microraptor* based on three features: the presence of mid-caudal vertebrae that are approximately three to four times longer (anteroposteriorly) than the anterior dorsal vertebrae; strongly recurved and slender pedal ungual with prominent flexor tubercle; extremely long metatarsal V (although not bowed like in IVPP V12330) [[1](#_ENREF_1),[2](#_ENREF_2)].

**References**

1. Xu X, Zhou Z, Wang X, Kuang X, Zhang F, et al. (2003) Four-winged dinosaurs from China. Nature 421: 335-340

2. Turner AH, Makovicky PJ, Norell MA (2012) A review of dromaeosaurid systematics and paravian phylogeny. Bulletin of the American Museum of Natural History 371: 1-206.
